# Multilevel factors influencing colorectal cancer screening adherence: A systematic literature review

**DOI:** 10.1371/journal.pone.0342184

**Published:** 2026-02-03

**Authors:** Nur Fadhilah Zubair, Azmawati Mohammed Nawi, Mohd Rohaizat Hassan, Amsyar Daud, Pongdech Sarakarn, Rosnah Sutan

**Affiliations:** 1 Department of Public Health Medicine, Faculty of Medicine, Universiti Kebangsaan Malaysia, Cheras, Kuala Lumpur, Malaysia; 2 University of Cyberjaya, Cyberjaya, Selangor, Malaysia; 3 Borneo Medical and Health Research Centre, Faculty of Medicine and Health Sciences, University Malaysia Sabah, Kota Kinabalu, Sabah, Malaysia; 4 Department of Emergency Medicine, Faculty of Medicine, Universiti Kebangsaan Malaysia, Cheras, Kuala Lumpur, Malaysia; 5 Department of Epidemiology and Biostatistics, Faculty of Public Health, Khon Kaen University, Khon Kaen, Thailand; Hue University of Medicine and Pharmacy, VIETNAM

## Abstract

**Introduction:**

Colorectal cancer (CRC) is one of the leading causes of cancer-related morbidity and mortality worldwide. Although early detection through screening significantly reduces mortality, adherence to recommended screening remains suboptimal. This systematic review examines the multilevel factors influencing CRC screening adherence, and integrates the findings within the Socio-Ecological Model to provide a structured analytical framework.

**Methods:**

A systematic search was conducted across PubMed, Scopus, and Web of Science for studies published between 2000 and 2024 that employed multilevel modeling to examine CRC screening behavior. Eligible studies involved average-risk adults and reported both individual- and contextual level determinants of screening adherence. Studies focusing exclusively on clinical predictors or non-screening outcomes were excluded. Risk of bias was assessed using the Joanna Briggs Institute Critical Appraisal Tools. A narrative synthesis was performed to identify key individual, interpersonal, community, institutional, and policy-level determinants of CRC screening adherence.

**Results:**

Nine studies met the inclusion criteria, predominantly from high-income settings. At the individual level, older age, female sex, higher socioeconomic status, and health insurance coverage were consistently associated with greater screening adherence. Community factors such as neighborhood socioeconomic status and healthcare accessibility, influenced screening behavior, while institutional elements included system structures and service availability. Policy-level determinants, such as national health insurance and national screening guidelines, were less frequently examined but demonstrated measurable effects. Despite heterogeneity in populations, synthesis within the Socio-Ecological Model highlighted the interconnected nature of these determinants and emphasized the need for multilevel interventions targeting individual, social, and structural determinants.

**Conclusion:**

This review emphasizes the importance of addressing CRC screening behavior through a multilevel perspective that incorporates individual, social, and structural determinants. Future research should explore these determinants in low- and middle-income settings and assess the effectiveness of integrated multilevel interventions in improving CRC screening adherence.

## Introduction

Colorectal cancer (CRC) represents a significant global health challenge, ranking among the leading causes of cancer-related morbidity and mortality globally. According to Global Cancer Observatory, CRC accounted for approximately 10% of all cancer cases and nearly 9.4% of all cancer-related deaths in 2020, making it the third most diagnosed cancer and the second leading cause of cancer mortality [1]. Projections suggest a significant rise in CRC mortality globally by 2035, with mortality anticipated to increase by 60% for colon cancer and 71.5% for rectal cancer from 2013 to 2025 [2]. Although variations in CRC trends exist among countries, the overall increase in incidence and mortality is primarily linked to aging populations and evolving risk factor exposure such as increasing prevalence of obesity, smoking, and diets characterized by high levels of processed meats and artificial sweeteners [2]. These trends call for urgent and effective prevention strategies.

Early detection via CRC screening significantly decreases mortality and morbidity by facilitating the identification and treatment of precancerous lesions and early-stage cancers. Screening programs have demonstrated effectiveness, evidenced by significant reductions in colorectal cancer-related mortality among populations with high screening participation [2,3]. Adherence to standard CRC screening protocols considerably increases the sensitivity and effectiveness of these programs, ensuring timely intervention and reducing the incidence of advanced-stage CRC [4,5]. Despite evidence supporting the benefits of screening, adherence rates remain suboptimal. Community-based surveys in the United States report wide variations, ranging from 13–55%, while more recent national estimates indicate that approximately 71.6% of adults aged 50–75 years are up to date with CRC screening [6]. Similar variations are observed in Asia-Pacific countries such as 21% in South Korea and 62.9% in Thailand [7].

Colorectal cancer screening adherence is influenced by a multitude of factors, including demographic factors such as age, ethnicity and socioeconomic status, healthcare system factors comprising of screening accessibility and affordability, as well as physician-patient relationship [8]. Psychosocial determinants such as knowledge, risk perception, and attitudes are also important predictors of persistent adherence to CRC screening [8]. A comprehensive strategy is needed to address the multilevel disparities, enhance accessibility, and improve physician-patient communication to facilitate higher rates of colorectal cancer screening adherence.

Given the complex interplay of factors influencing CRC screening adherence, multilevel analysis has become increasingly prominent in statistics and research because it allows simultaneous examination of individual and contextual-level variability. The use of linear mixed models offers a significant advantage due to their capacity to effectively handle hierarchical data structures, by integrating fixed effects that denote population-level parameters and random effects that address individual-level variability, such as individuals within communities or healthcare systems [9,10]. Linear-mixed models partition variance into fixed and random components, enabling more accurate estimation of population-level effects while accounting for clustering within hierarchical data structures. [9,11]. Furthermore, multilevel analysis provides enhanced adaptability in contrast to conventional linear models, particularly when addressing unbalanced data or datasets that contain missing values [9]. In public health research, this approach has demonstrated empirical value. For example, when an epidemiological study evaluates health outcomes across various geographical regions, linear mixed models effectively address variations stemming from individual traits and regional influences. A recent analysis of cancer screening determinants in selected European countries demonstrated that macro-level healthcare system characteristics and social expenditures modify the inequalities observed in pap smear and mammography uptake [12,13]. This analytical approach yields more detailed insights than simpler models that overlook hierarchical structures, and supports the use of multilevel models for examining determinants of cancer screening behavior [14].

Hence, this review aimed to systematically analyze the factors influencing colorectal cancer screening adherence using multilevel modeling. Additionally, this review seeks to map the existing evidence within a theoretical framework and identify gaps in the understanding of cancer screening adherence.

## Methodology

PCC Framework was used to outline the key elements of this review as illustrated in Table 1, which led to the main research question “What is the available evidence of predictors that can influence colorectal cancer screening behavior among average-risk populations, as identified in studies using multilevel analysis approaches?”. This review uses the term CRC screening behavior as an umbrella term encompassing study-specific operationalization’s of screening adherence, including initial adherence to guideline (ever having been screened), timely adherence, and longitudinal adherence (repeated or interval-based participation), consistent with methodological guidance on cancer screening measurement [15].

**Table 1:** PCC framework.

**Population**	Average-risk populations that are eligible for colorectal cancer screening based on national guidelines
**Concept**	Colorectal cancer screening behavior – encompassing initial adherence and longitudinal adherence to screening recommendations.
**Context**	Multilevel analysis utilizing hierarchical data

### Protocol registration and study selection process

This systematic review was registered with the International Prospective Register of Systematic Reviews (PROSPERO) under registration number CRD42024578424. The review protocol adhered to the guidelines of the Preferred Reporting Items for Systematic Reviews and Meta-Analyses Protocol (PRISMA-P) [16].

The first phase of the review involved identifying relevant keywords based on the PCC framework formulation. A systematic search was then conducted using a combination of keywords and Boolean operators, such as “colorectal cancer”, “screening adherence”, “multilevel analysis”, and their synonyms. The search strategy was tailored for each database as summarized in Supplementary Table 2 in S2 File. These comprehensive searches were conducted in 3 main databases – PubMed, Scopus and Web of Science. These databases were chosen due to their comprehensive coverage and indexing quality of high-quality, peer-reviewed literature in relevance to the review topic. Reference lists of eligible studies and relevant review articles were also hard-searched to maximize yield. The search covers literature published from January 2000 to the most recent search record in October 2024, reflecting the rise of multilevel analysis approaches in public health research. No language restrictions were imposed during the sourcing strategy.

**Fig 1:**
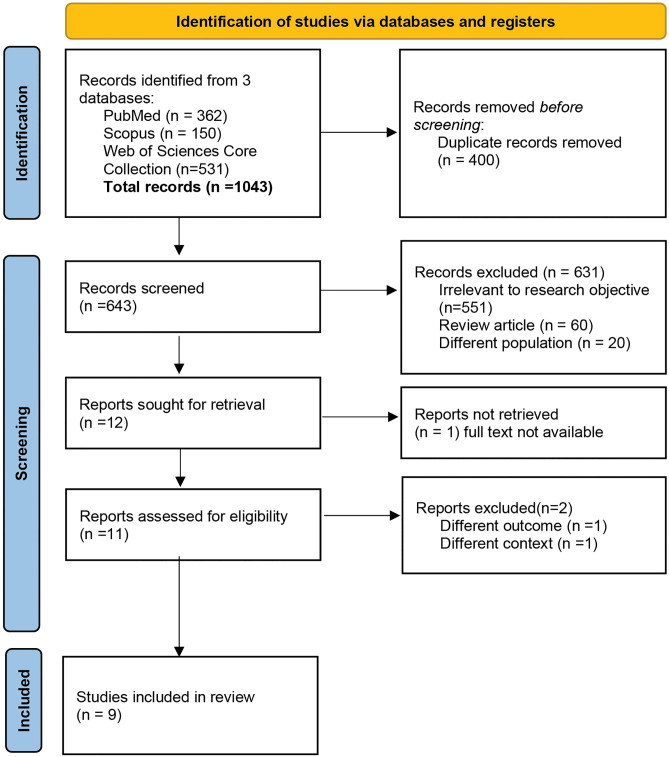
PRISMA flow diagram of the study selection process.

Studies that examined colorectal cancer screening behavior among average-risk populations were included to address the research question and objectives of this review. The inclusion criteria include quantitative and mixed methods studies to capture contextual factors influencing colorectal cancer screening behavior. Review articles, conference proceedings were excluded, allowing the focus on empirical research to gather new knowledge and synthesizing. Additionally, studies that focus on a population with high risk for colorectal cancer such as genetic predisposition of Lynch Syndrome were excluded. Only studies employing multilevel or hierarchical modelling techniques were included to ensure methodological consistency. The study selection process followed the PRISMA guideline and is summarized in the PRISMA flowchart indicated in Fig 1. The search results identified through database searches were imported into the reference manager EndNote X8 where they were screened for duplicates. Three reviewers (NFZ, AMN, MRH) pre-screened the title and abstracts, consulting another reviewer (PS) when any uncertainties arise. Full-text screening, assessment of eligibility, and quality appraisal of the shortlisted articles were then carried out independently by all reviewers. The final included studies were evaluated for quality using the JBI Critical Appraisal Checklist for Analytical Cross-Sectional Studies. Additionally, we appraised the applications of multilevel modeling across final selected studies. These findings are detailed in Supplementary Table 3 in S3 File.

### Data extraction and synthesis of results

Authors jointly developed and agreed upon a standardized data extraction form in Microsoft Excel. Extraction was performed independently by one reviewer and verified by a second. Following the completion of data extraction, a content analysis of the extracted data was performed. The studies were classified according to the predetermined criteria. The synthesis of quantitative findings aimed to identify associations between explanatory variables or determinants and colorectal cancer screening behavior across studies that utilizes multilevel modelling analysis. The variables were summarized according to their statistical significance, indicating positive or negative associations with CRC screening behavior.

## Results

The initial search conducted across three databases resulted in a cumulative total of 1,043 records. Following the elimination of 400 duplicate records, 643 unique records were retained for screening. Throughout the screening process, a total of 631 records were excluded due to the following reasons: irrelevance to the research objective (n = 551), classification as review articles (n = 60), and focus on a different population (n = 20). A total of 12 reports remained for retrieval. One report was inaccessible due to the lack of full text and two were excluded for addressing a different outcome. Nine studies were ultimately included in the final review.

### Characteristics of included studies

Table 2 presents a summary of the articles included in this review. This review encompasses studies published from 2008 to 2022. The studies included were all conducted in high-income countries, comprising five studies from the United States [17–21], two from France [22,23], one from Switzerland [24], and one from Italy [25]. These studies encompass a diverse range of populations, including Vietnamese Americans in Philadelphia, a state sample in France, and a national sample from Switzerland. Population studies typically encompass individuals aged 50 years and older or adhere to the specific age guidelines for colorectal cancer screening established by respective countries.

**Table 2:** Characteristics of included studies.

No	Author, year	Country	Title	Study objective	Study design and setting	Study population and sample size (n)
**1**	[17]	United States	Examining multilevel neighborhood socioeconomic characteristics associated with colorectal cancer screening in Vietnamese Americans residing in Philadelphia County	To examine the impact of the built environment and neighborhood environmental factors on colorectal cancer (CRC) screening behavior among Vietnamese Americans residing in Philadelphia County.	The study utilized a community-based participatory research approach for recruitment and data collection. The participants were part of a clustered randomized culturally appropriate, theory- and evidence-based multilevel colorectal cancer intervention.	Vietnamese American aged 50 years and older (n = 517)
**2**	[24]	Switzerland	Variation of colorectal, breast and prostate cancer screening activity in Switzerland: Influence of insurance, policy and guidelines	To analyze the geographic variation of colorectal, breast, and prostate cancer screening utilization in Switzerland and the influence of available guidelines and different modifiers of access.	Cross-sectional – utilizing administrative insurance claims data from major insurer in Switzerland	Eligible population for each cancer screening type, for colorectal was 50–69 years old (n = 1.2 million)
**3**	[22]	France	Predictive factors for non-participation or partial participation in breast, cervical and colorectal cancer screening programs	To describe patterns and rates of non-participation or partial participation in combinations of screening programs for breast, cervical, and colorectal cancers among French women aged 50–65 years	Cross-sectional using retrospective data from screening center	Female resident aged between 50 and 74 years (n = 102,219)
**4**	[25]	Italy	Geospatial analysis of the influence of family doctor on colorectal cancer screening adherence	To investigate the determinants of colorectal cancer (CRC) screening adherence in the regional population, focusing on the influence of individual factors and clustering factors corresponding to health service components, such as family doctors (FDs) and health districts.	Cross-sectional utilizing state government data	People aged 50–74 years who were invited over three screening rounds (n = 333,843)
**5**	[18]	United States	Geographic and population-level disparities in colorectal cancer testing: A multilevel analysis of Medicaid and commercial claims data	To understand differences in relative rates of colorectal cancer (CRC) testing across Oregon’s Medicaid and commercially insured populations	Retrospective analysis of insurance claim data and area health resource data	Health insurance beneficiaries comprising both Medicaid and commercial insurance (n = 64711)
**6**	[20]	United States	Associations between contextual factors and colorectal cancer screening in a racially and ethnically diverse population in Texas	To examine the associations between socioeconomic measures and adherence to colorectal cancer screening	Cross-sectional, utilising state-level health survey and state census tract data	Individual aged 50–74 years old (n = 1720)
**7**	[23]	France	Predictors of adherence to repeat fecal occult blood test in a population-based colorectal cancer screening program	To identify the proportion of people who completed repeat fecal occult blood testing (FOBT) among those eligible for all the first three rounds of colorectal cancer screening, and to identify both individual and contextual factors associated with adherence to repeat screening.	Retrospective analysis using administrative insurance claims data	Recipients of French statutory Health Insurance, aged between 50–74 years between September 2007 and September 2010 (n = 61,386)
**8**	[21]	United States	Geographic variation and effect of area-level poverty rate on colorectal cancer screening	To analyze the geographic variation and the impact of area-level poverty rate on colorectal cancer screening among individuals aged 50 or older in Missouri.	Cross sectional survey and geographical mapping using postal code	Individual aged 50 or older, residing in Missouri (n = 3022)
**9**	[19]	United States	Multilevel predictors of colorectal cancer screening use in California	To examine the geographic variation in colorectal cancer (CRC) screening	Cross-sectional utilizing data from state survey and government health records	Individual aged 50–84 years old residing in California (n = 20,626)

### Measures of colorectal cancer screening behavior

In this review, the term colorectal cancer screening behavior is used as an umbrella construct that encompasses various operational definitions of adherence reported in the included studies. These can be broadly categorized into three sub-definitions where: (1) Initial adherence to CRC screening, defined as having undergone any CRC screening modalities- regardless of time interval, (2) Timely adherence, defined as undergoing screening within the recommended time frame, such as annual FOBT, colonoscopy every 10 years and sigmoidoscopy every 5 years; and (3) Longitudinal adherence, defined as repeated or regular participation in screening over time. Specifically, four studies reported adherence as participation [17,18,24,25], three studies used timely adherence based on modality specific intervals [19–21], and two studies focused on longitudinal adherence [22,26]. Despite minor variations in the definition of CRC screening adherence, the prevalence of adherence across the reviewed literature ranges from 22.05% to 60.3% among the studied populations.

### Factors influencing CRC screening behavior

The determinants of CRC screening behavior were categorized into multiple levels based on the Socio-Ecological Model (SEM), a well-established framework for understanding health behaviors in a broader context. The SEM, originally introduced by Bronfenbrenner (1986), recognizes that individual behavior is shaped by the complex interplay of personal, interpersonal, community, institutional, and policy-level influences [27]. The Socio-Ecological model (SEM) outlines multiple levels of impacts on a person’s action, starting from the closest microsystem to the wider macrosystem, and including changes over time in the chronosystem. The findings of this review are integrated into the SEM framework to provide a systematic framework for assessing the factors influencing CRC screening adherence. Applying this model enables us to examine how personal factors interact with external environmental influences, ensuring a more holistic understanding of screening behaviors. Table 3 summarizes the existing evidence of factors influencing CRC screening behavior adapted into the SEM model.

**Table 3:** Summary of factors influencing CRC screening behavior adapted into Socio-Ecological Model (SEM).

Author (Year)	Concept definition	Proportion of the population that adheres to CRC screening	Factors influencing colorectal cancer screening behavior				
			Individual level	Interpersonal level	Institutional level	Community level	Policy level
Initial adherence to CRC screening: Studies defining adherence as any participation or uptake of screening modalities.							
**[17]**	Individual uptake of any CRC screening modality	22.5% that had prior history to any CRC screening tests	-Older age 65 and above (+)	NA	-Distance to nearest Healthcare facilities	-Ethnically dense neighborhood (Asian) (-) -Social deprivation index -Walk score	NA
**[24]**	Individuals who utilize colonoscopy test	57% of the eligible population would have received colonoscopies	-Age (+) -Presence of comorbidities (+)	-Participation in care plans (+)	NA	-Living in area with higher purchasing power (+) -Urban (+)	-Insurance policy, higher coverage (+) -Supplementary insurance (+) -Hospital care insurance (+)
**[25]**	Individuals that adhere to the first screening invitation for colonoscopy	40.2% adhered to the first invitation for CRC screening	-Foreign-born (-) -Youngest and oldest age group (-) -Male (-)	NA	-Presence of Family Doctor practices (+)	-District by geographical	NA
**[18]**	Individual received any type of CRC screening modality during the 4-year period study	42.2% of the beneficiaries’ evidence of utilizing CRC screening test	-Female (+) -Commercial insurance (+)	NA	-Primary care engagement (+) -High family doctor: patient ratio (-) -Distance to endoscopy facility -Access to primary care (+)	-Urban (+) -Higher socioeconomic deprivation (-)	NA
Timely adherence: Studies that define adherence based on screening within recommended time frames (e.g.,: annual FOBT, 10-year colonoscopy)							
**[20]**	Individual was considered adherent if reported any utilization of CRC screening test within time frame	58.0% of the sample was adherent to any timely CRCS test	-Hispanic (-) -Foreign-born (-) -Younger age 50–59 years (-) -Single, separated, divorced (-) -Unemployed (-) -No health insurance (-) -High family income (+)	NA	NA	-Area level unemployment (-)	NA
**[19]**	Individuals that were up-to date with any CRC screening modalities within the time frame	60.3% aged 50–84 years were reported being up-to-date with at least one of the screening modalities.	Non-Hispanic/White (+) Married (+) Educated (+) US-born (+) Insured (+)	NA	Primary care shortage (-)	Safe neighborhood (+) No food insecurity (+)	NA
**[21]**	Individuals that had a FOBT done in past year and/or having sigmoidoscopy or colonoscopy in past five years	51.8% of individuals aged 50 years and above	Retired (+) Higher BMI (+) Anxiety/ depression history (+) Low education (-) Mid income household (-) No insurance (-)	NA	Access to primary care (-) No primary care physician (-) No routine checkup (-)	NA	NA
Longitudinal adherence: Studies defining adherence as repeat or sustained participation in CRC screening							
**[23]**	Individuals that have a potential repeat screening within the recommended timeline	38.8% compliant participants that adhere to repeat screening.	-Male (-) -Self-employed (-)	-Invitation via mail (-) -Invitation via general practitioner (+)	NA	-Living in deprived area (-) -Rural/ urban weak	NA
**[22]**	Individuals that were up-to-date with CRC screenings based on modalities	24.7% adhere to colorectal cancer screening	-Younger age 50–55 years (-) -Self-employment (-) -Workplace insurance (-)	NA	NA	-Lower Socioeconomic status (-)	NA

Footnote:

(+) significant positive association.

(-) significant negative association.

(no symbol) – no significant association.

NA – not applicable.

Within the individual level, socio-demographic factors, such as age, gender, race/ethnicity, socioeconomic status, and insurance coverage, were identified as significant determinants of CRC screening behavior. Age significantly predicted screening adherence, with individuals over 65 years demonstrating higher rates, in contrast to those aged 50–55 [22,28]. Gender differences were noted, with males showing lower screening adherence than females [25] Ethnic disparities were evident, with foreign-born individuals and minority populations exhibiting lower CRC screening rates attributed to barriers like language, cultural differences, and healthcare access [20,25]. Socioeconomic status significantly influenced screening behavior, as individuals from lower-income households and the unemployed exhibited lower rates of adherence [20]. Additionally, personal health insurance or workplace-related coverage was positively associated with adherence to CRC screening [19,22].

The interpersonal level focuses on the influence of social networks, family support, and healthcare provider interactions on screening behavior. Only three of the included studies in this review have examined these interpersonal aspects where participation in a structured care plan and having access to a consistent family doctor were correlated with increased adherence [21,25]. Importantly, screening invitations issued by general practitioners resulted in higher adherence rates compared to mail invitations [26].

Individuals in socioeconomically disadvantaged areas, high-unemployment communities, and rural regions exhibited lower screening rates in CRC screening than those in affluent urban neighborhoods [20,22,26]. Access to primary care, screening services, and screening facilities consistently predicted CRC screening adherence. Individuals residing near endoscopic facilities or with higher primary care provider-to-patient ratios are more likely to engage in CRC screening [18].

**Fig 2:**
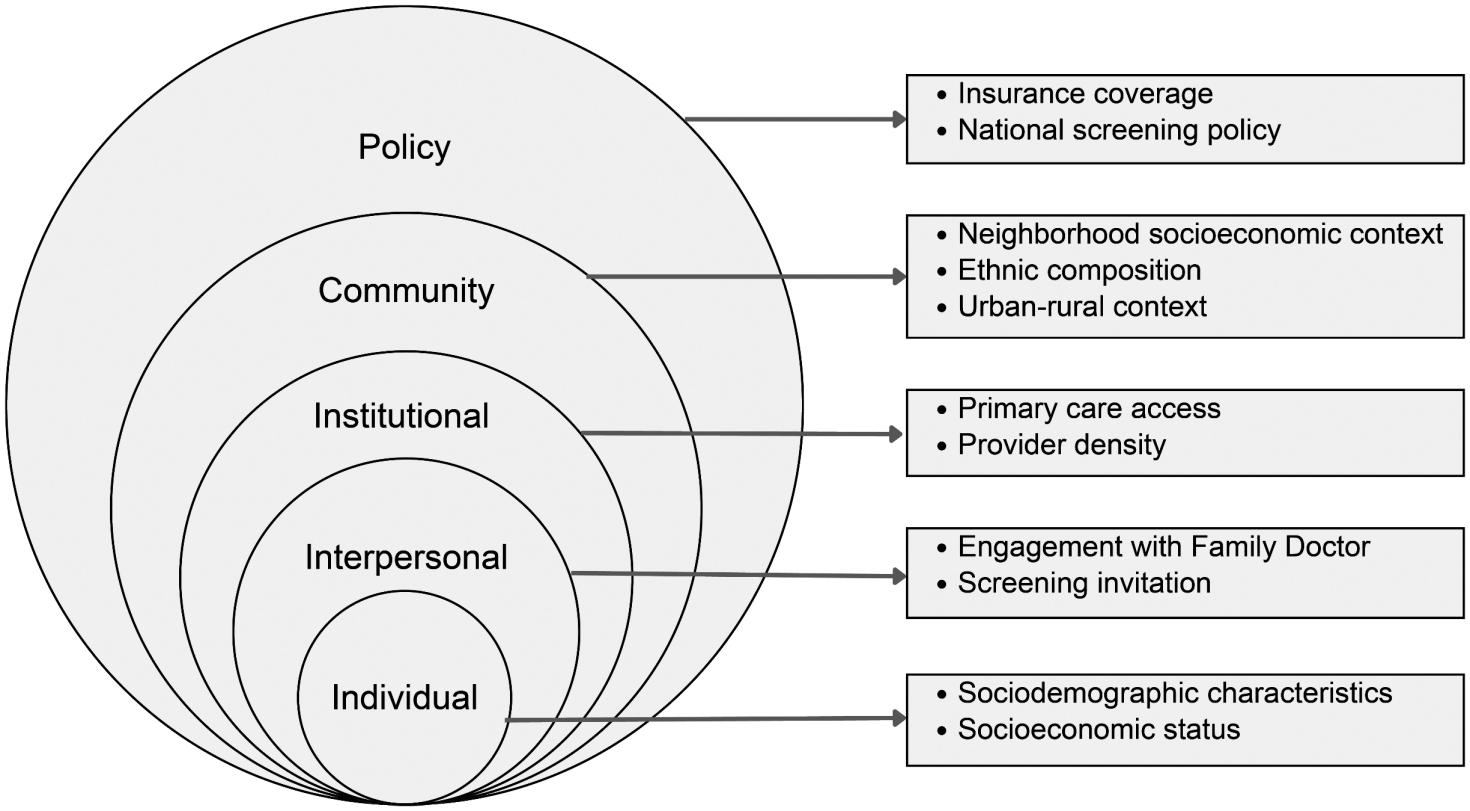
Summary of multilevel determinants of colorectal cancer screening adherence mapped to the Socio-Ecological Model (SEM).

Policy-level determinants were the least explored in the included studies, with only one study identifying insurance coverage as a determinant, where finding suggest that financial accessibility and system-level policies significantly influence screening adherence [24]. This limited representation suggests a gap in exploration of macro-level policy variables. Integrating these findings within the socio-ecological model (SEM) framework, as illustrated in Fig 2, offers a thorough understanding of CRC screening behaviors across individual, social, organizational, and structural domains allowing for identification of various points for public health interventions.

## Discussion

A key finding of this review is the significant variability in the reported screening adherence rates, primarily due to differing operational definitions of adherence across studies. As highlighted in prior work, heterogeneity in the definition of cancer screening adherence presents a significant challenge for evidence synthesis and limits comparability across contexts [15], emphasizing the need for enhanced definitional clarity in future research. At the individual level, socio-demographic factors are most commonly discussed determinants of colorectal cancer screening behavior. Older adults showed higher adherence to CRC screening than younger individuals, likely due to a greater perceived susceptibility to cancer and increased healthcare engagement [29,30]. Women showed a higher inclination to participate in CRC screening, likely due to increased health consciousness and a tendency to seek preventive care [29,31]. Married individuals likely benefit from enhanced social support and encouragement for screening adherence.

However, beyond these socio-demographic factors, an individual’s behavior toward cancer screening is also influenced by their psychological and sociocultural values [32–34]. Belief systems, encompassing attitudes, knowledge, and perceived susceptibility to colorectal cancer, are powerful drivers of screening decisions yet often less quantified. Moreover, individual’s health literacy is increasingly recognized as a significant predictor of health-seeking behavior, including adherence to screening where the extent of an individual’s understanding and awareness about CRC critically impacts their participation in screening tests [35]. This review's emphasis on quantitative multilevel studies results in a limited representation of these complex individual-level determinants, which may not adequately capture these nuanced constructs. Future reviews may include a broader approach to enhance our comprehension of the influence of cultural context and individual cognition on screening adherence. At the interpersonal level, patients involvement with the healthcare system and their relationships with healthcare providers have become significant determinants of adherence to colorectal cancer screening. [36] illustrated that the method of patient invitation and their active involvement in treatment planning are essential factors influencing CRC screening rates. Primary care providers serve as essential frontline clinicians in establishing and nurturing a trusting relationship between patients and the healthcare system. This trust is crucial for collaborative decision-making, which can profoundly influence an individual’s screening practices. Patients who feel empowered and engage actively in their care are more likely to comply with suggested CRC screening recommendations [24]. Moreover, the characteristics of the patient-provider contact, including the communication tone, the level of patient-centeredness, and the delivery of customized education and support, might affect the patient’s impressions of screening and eventually impact their adherence in screening. Interpersonal-level intervention aimed at boosting patient-provider collaboration, improving shared decision-making, and cultivating a supportive healthcare environment may be especially successful in promoting adherence to CRC screening.

Beyond individual and interpersonal dynamics, this review highlights the significance and interconnected impact of institutional and community factors on cancer screening behavior. Neighborhood characteristics, urbanicity, and ethnic composition at the community level significantly influence screening adherence; individuals in socioeconomically disadvantaged or rural areas frequently encounter compounded barriers to screening [17,18,22,24,26]. Community-level disparities are often intensified by institutional factors, such as restricted access to primary care, lack of screening facilities and insufficient support from the healthcare system [17,19,25,36]. Transportation emerges as a critical factor influencing adherence to cancer screening, particularly in rural populations who face substantial barriers such as long travel distances, unreliable transportation options, and procedural requirements such as the need for escorts, which collectively limit access to CRC screening [37,38]. Therefore, effective interventions must integrate community-based strategies, such as community-based transportation solutions and targeted community outreach programs to mitigate these challenges, creating safer and more equitable access to screening facilities [39].

Community support, which can range from a customized program enabling access for specific groups of the population, to a broader community-based awareness program, has been shown to improve cancer screening participation rates [40,41]. A specific community-based participatory research also found that collaboration between community members and health professionals is associated with higher CRC screening adherence in underserved communities [40]. This is also supported in a 4 arm randomized controlled trial, where the combination of community outreach with in-clinic one-to-one education with health professionals tripled CRC screening uptake as compared to community outreach alone or in-clinic patient education alone [42]. This finding further supports the notion that community-based strategies must go in line with institutional improvements, such as the implementation of evidence-based screening guidelines, robust resources or patient education and navigation, and optimal integration of timely reminders into electronic health records. Recognizing the complex relationship between broader community context and healthcare delivery system calls for a synergistic approach in improving equitable cancer screening adherence.

Policy-level factors are notably under-researched among the multilevel determinants in this review, representing a significant gap in the current literature. However, current existing evidence consistently highlights the importance of policies, particularly comprehensive insurance coverage for screening services, in promoting adherence and reducing disparities [18,20,24]. As a result, comprehending and putting into practice strong policy frameworks are crucial complements to interventions at other socioecological levels, ultimately encouraging more widespread and equitable CRC screening adherence. Although the SEM framework serves as a valuable framework for categorizing influences on CRC screening adherence, the included multilevel research predominantly focuses on individual and institutional factors, with interpersonal and structural dimensions, especially policy, being less frequently addressed. The underrepresentation of interpersonal-level determinants may be attributed to methodological limitations, particularly the challenges in operationalizing social constructs within quantitative multilevel frameworks. However, the scarcity of policy-level factors likely represents a substantive gap in the literature, highlighting the necessity for future research to more clearly incorporate policy-level determinants.

Following the synthesis, it became evident that several determinants – such as access to primary care, insurance coverage, and provider-to-patient ratio, transcend strict categorical limits within the framework. For example, provider communication, while inherently interpersonal, is embedded within the broader institutional-level systems. Insurance may be shaped by policy-level decisions, yet its impact is mediated by individuals’ affordability or socioeconomic status. This overlap highlights the interdependence of SEM domains and supports prior arguments that the multilevel health behavior framework should be applied dynamically instead of hierarchically [43,44]. Interactions between domains are methodologically limited as reflected in Supplementary Table 3 in S3 File, where most studies used two-level hierarchical models without specifying cross-level interactions, limiting a more comprehensive evaluation of dynamic interdependencies.

In summary, CRC screening adherence is influenced by a complex interaction of multilevel factors, including individual attributes, interpersonal relationships, community context, and broader policy frameworks. Current screening programs need to enhance coordination by addressing contextual barriers such as financial strain, transportation insecurity, and health literacy that disproportionately impact vulnerable populations [45]. Integrated approaches that involve multilevel interventions such as community-based programs and population-targeted policy reforms are essential for improving CRC screening adherence [36]. This approach not only enhances the adherence in screening programs, but also aligns with the public health objective of reducing morbidity and mortality linked to CRC. Bridging this gap in understanding and implementing multilevel strategies are necessary for improving CRC outcomes and ensuring that all individuals, regardless of their background, have equitable access to screening services.

### Strengths and limitations

This systematic review provides a comprehensive analysis of multilevel factors influencing CRC screening behavior by utilizing studies that employ multilevel modeling approaches. One of its key strengths is its focus on a theoretical framework, the Socio-Ecological model, which allows for a structured understanding of individual, interpersonal, community, institutional, and policy-level influences. Additionally, by including studies from diverse populations and geographical settings, this review provides a broader perspective on factors affecting CRC screening adherence.

However, this review has several limitations. First, the included studies predominantly originate from high-income countries with established CRC screening programs. This limits the generalizability of findings to low- and middle-income settings, where CRC screening programs are often opportunistic or lacking structure altogether. Second, variations in CRC screening definitions across studies may introduce inconsistencies in outcome measurement, affecting comparability. Third, discrepancies in the reporting of essential multilevel parameters such as intraclass correlation coefficient (ICC) and variance components, which limits direct comparison of multilevel effects. Finally, this review’s focus on quantitative multilevel studies, while methodologically justified, may have excluded representation of psychological, cultural, and belief-related factors that are more commonly explored in qualitative research.

## Conclusion

This review highlights the complexity of CRC screening behavior, demonstrating the necessity for interventions targeting various levels. Individual characteristics like age, gender, and socioeconomic status significantly affect screening adherence, while community and institutional factors, including healthcare accessibility and social support, also influence adherence. Policy-level factors, especially insurance coverage, are crucial for equitable access to CRC screening services. Future research should prioritize the inclusion of underexplored SEM domains found in this review, particularly interpersonal and policy-level determinants. Future research should also examine the applicability of these findings in low- and middle-income contexts and assess longitudinal trends in screening behaviors. Public health interventions must be designed with an understanding of the interconnections among individual, societal, and structural determinants. Incorporating multilevel evidence into program development can enhance the precision and sustainability of programs aimed at increasing CRC screening rates, particularly among populations encountering contextual and systemic barriers.

## Supporting information

S1 File PRISMA 2020 checklist. (DOCX)

S2 File Search string. (DOCX)

S3 File Critical appraisal of included studies. (DOCX)

S4 File Extracted dataset of included studies. (XLSX)
