# Supplementary material for: Multilevel factors influencing colorectal cancer screening adherence: A systematic literature review
Source: PLoS One. 2026 Feb 3;21(2):e0342184. doi: 10.1371/journal.pone.0342184 (PMC12867220; doi:10.1371/journal.pone.0342184)
Supplement: S2 File — (DOCX) [file pone.0342184.s002.docx]

**Supplementary Table 2: Search string**

| **Database** | **Keywords** |
| --- | --- |
| PubMed | ("multilevel"[Title/Abstract] OR "hierarchical"[Title/Abstract] OR "context*"[Title/Abstract] OR "multi-level"[Title/Abstract]) AND ("average risk"[Title/Abstract] OR "asymptomatic"[Title/Abstract] OR "population"[Title/Abstract]) AND ("colorectal cancer"[Title/Abstract] OR "colon cancer"[Title/Abstract] OR "rectal cancer"[Title/Abstract] OR "colorectal neoplasm"[Title/Abstract] OR "colon neoplasm"[Title/Abstract] OR "colon malignancy"[Title/Abstract]) AND ("screening"[Title/Abstract] OR "colonoscopy"[Title/Abstract] OR "sigmoidoscopy"[Title/Abstract] OR "iFOBT"[Title/Abstract] OR "fecal occult blood"[Title/Abstract]) |
| Scopus | ( ( average AND risk OR asymptomatic OR population ) AND ( colorectal AND neoplasms OR colorectal AND malignancy OR colon AND cancer OR rectal AND cancer ) AND ( screening OR colonoscopy OR sigmoidoscopy OR fobt OR fecal AND occult AND blood ) AND ( multilevel OR context* OR hierarchical OR nest* OR multi-level ) ) |
| Web of Science Core Collection | (((TS=(average risk OR asymptomatic OR population)) AND TS=(colorectal cancer OR colon cancer OR colorectal malignancy OR colon neoplasm OR rectal cancer)) AND TS=(screening OR colonoscopy OR sigmoidoscopy OR iFOBT)) AND TS=(multilevel OR multi-level OR hierarchical OR context*) |
