# Supplementary material for: Multilevel factors influencing colorectal cancer screening adherence: A systematic literature review
Source: PLoS One. 2026 Feb 3;21(2):e0342184. doi: 10.1371/journal.pone.0342184 (PMC12867220; doi:10.1371/journal.pone.0342184)
Supplement: S3 File — (DOCX) [file pone.0342184.s003.docx]

**Supplementary Table 3: Critical appraisal of included studies and Multilevel Modelling appraisal**

|  | Reference | Q1 | Q2 | Q3 | Q4 | Q5 | Q6 | Q7 | Q8 | Multilevel modeling appraisal | |
| --- | --- | --- | --- | --- | --- | --- | --- | --- | --- | --- | --- |
| 1 | [14] | Yes | Yes | Yes | Unclear | Yes | Yes | Yes | Yes | Generalized Linear Mixed Model with random effect for census tract, no ICC/VPC reported. Nesting rationale. |  |
| 2 | [21] | Yes | Yes | Yes | Yes | Yes | Yes | Yes | Yes | Multilevel logistic regression, random intercept with spatial component |  |
| 3 | [20] | Yes | Yes | Yes | Yes | Yes | Yes | Yes | Yes | Multilevel logistic regression (random intercept model), ICC reported 1.0% for colorectal cancer screening. |  |
| 4 | [22] | Yes | Yes | Yes | Yes | Yes | Yes | Yes | Yes | Multilevel logistic regression with random intercept and random slope, VPC reported, and MOR computed to quantify clustering |  |
| 5 | [15] | Yes | Yes | Yes | Yes | Yes | Yes | Yes | Yes | Multilevel logistic regression with random intercept. No ICC reported. |  |
| 6 | [16] | Yes | Yes | Yes | Unclear | Yes | Yes | Yes | Yes | Two-level random intercept logistic model, ICC reported. |  |
| 7 | [17] | Yes | Yes | Yes | Yes | Yes | Yes | Yes | Yes | Multilevel logistic regression (random intercept model), does not report ICC/VPC. Nesting rationale |  |
| 8 | [18] | Yes | Yes | Yes | Yes | Yes | Yes | Yes | Yes | Three‐level multilevel logistic regression, ICC reported |  |
| 9 | [19] | Yes | Yes | Yes | Yes | Yes | Yes | Yes | Yes | Three-level random intercept logistic model, ICC, MOR AND IOR computed. |  |

| Legend for Multilevel modelling appraisal |  |
| --- | --- |
| Strong rationale with ICC/VPC or MOR reported |  |
| Nesting rationale used however, no metrics reported |  |
| Multilevel modeling used without rationale or metrics |  |
